# Bonding of Glass-Ionomer Cement and Adhesives to Silver Diamine Fluoride-treated Dentin: An Updated Systematic Review and Meta-Analysis

**DOI:** 10.3290/j.jad.b2701679

**Published:** 2022-03-01

**Authors:** Tatiana Tambara Fröhlich, Graziela Botton, Rachel de Oliveira Rocha

**Affiliations:** a Graduate Student, Graduate Program in Dental Science, Federal University of Santa Maria, RS, Brazil. Conceived the idea and study design, performed the literature search, extracted data, performed the meta-analysis, wrote the manuscript.; b Professor, Postgraduate Program of Pediatric Dentistry, Ingá University Center-Uningá, Santa Maria, RS, Brazil. Contributed substantially to discussion.; c Associate Professor, Department of Stomatology, Federal University of Santa Maria, Brazil. Conceived the idea and study design, contributed substantially to discussion, proofread the manuscript.

**Keywords:** bond strength, adhesion, glass-ionomer cement, adhesive, silver diamine fluoride

## Abstract

**Purpose:**

To evaluate through a systematic review and meta-analysis the bonding performance of adhesive materials to silver diamine fluoride (SDF)-treated dentin.

**Materials and Methods:**

Studies located in PubMed, Web of Science, LILACS, and Scopus up to September 2020, which compared the bond strength of adhesives (AD) or glass-ionomer cement (GIC) to SDF-treated and untreated (control) dentin were included. Mean differences were estimated separately by material and dentin condition (sound or caries-affected), with a random-effects model, at a 5% significance level.

**Results:**

Twenty-two studies, including 11 new studies not included in our previous systematic review, met the eligibility criteria, and 21 studies were considered in the meta-analyses. SDF dentin pretreatment did not influence the bonding of GIC (Z = 0.53; p = 0.60), independent of dentin condition. SDF treatment significantly impaired the bonding of AD (Z = 2.43; p = 0.01). A rinsing step after SDF eliminated this effect in sound dentin (Z = 1.82; p = 0.07) and increased the bond strength to caries-affected dentin (Z = 2.14; p = 0.03).

**Conclusion:**

SDF pretreatment does not influence the bond strength of GIC. A rinsing step after SDF application can improve the bond strength of AD to caries-affected dentin.

Silver diamine fluoride (SDF) has been considered the most effective non-invasive treatment for carious lesions, especially in primary teeth.^5^ SDF has bactericidal properties, inhibits demineralization, and promotes the remineralization of demineralized dentin.^56^ Also, it inhibits collagenases (matrix metalloproteinases and cysteine cathepsins) and protects dentin collagen from destruction.^30,31^

Due to these properties, SDF has a potential application as an adjunct to restorative treatment to prevent recurrent caries lesions.^26,53^ In vitro studies show that SDF dentin treatment prior to placing restorations provides greater resistance to the development of new lesions when submitted to cariogenic challenge.^32,36,57^ However, a silver phosphate layer was formed on the SDF-treated dentin surface,^40^ and silver particles extend into the dentinal tubules, totally or partially obstructing them,^22^ which could impair the adhesion of restorative materials.

A previous systematic review and meta-analysis^11^ showed that SDF pretreatment does not negatively influence the bond strength of glass-ionomer cement to dentin, but can impair the adhesive bond strength. The rinsing step after SDF application eliminates this adverse effect. However, few studies considered caries-affected dentin at the time of the review, and a separate meta-analysis with these data could not be performed. Worse results can be expected for caries-affected dentin, as chemical and morphological differences (eg, lower mineral content^1^ and increased porosity of intertubular dentin^33^) jeopardize bonding.^18^ Moreover, it is essential to consider that more silver precipitated onto demineralized than sound dentin.^22^

Interest in using SDF as a dentin pretreatment has increased since the publication of the systematic review mentioned above.^11^ Several studies have been published recently which evaluated this effect on caries-affected dentin,^3,9,19,34,46,49,50^ a relevant substrate in daily clinical practice. Some authors found that prior SDF application on caries-affected dentin does not diminish bond strength,^19,50^ or can even improve it.^9,46^ In contrast, other studies showed a significant reduction in bond strength.^3,26^ Therefore, this study aimed to update the systematic review and meta-analysis on the influence of silver diamine fluoride on the bonding performance of direct restorative materials to sound and caries-affected dentin. The null hypothesis tested was that SDF pretreatment does not influence the bond strength of glass-ionomer cement and adhesives regardless of the dentin condition – sound or caries-affected.

## Materials and Methods

This systematic review was written following the Preferred Reporting Items for Systematic Reviews and Meta-Analyses (PRISMA) Statement.^38^ The literature approach and search strategy were developed based on the following PICO (participant/problem, intervention, comparator, and outcome) question: does prior application of silver diamine fluoride influence the bond strength of direct restorative materials to sound and caries-affected dentin? The direct restorative materials (glass-ionomer cement and adhesives) was the “participant/problem”, prior silver diamine fluoride application was the “intervention”, no previous application was the “comparator”, and the bond strength was the “outcome”. The systematic review protocol was not previously registered; it can be accessed by contacting the authors.

### Search Strategy

A comprehensive literature search was undertaken through the electronic databases PubMed/MEDLINE, ISI Web of Science, Scopus, and LILACS to identify literature up to September 2020, with no language or publication year limits.

The subject search used a combination of controlled vocabulary and text words based on the search strategy developed for the PubMed/MEDLINE database as follows: ((((((((((((bond strength) OR microtensile) OR micro shear) OR tensile) OR Tensile Strength[MeSH Terms]) OR tensile strength) OR shear) OR shear strength) OR Shear Strength [MeSH Terms])) AND (composite resins[MeSH Terms]) OR composite resins) OR composite resin*) OR resin composite* OR Adhesives[MeSH Terms] OR adhesive* OR adhesion OR adhesive* OR Dental Bonding [MeSH Terms] OR dental bonding OR Dentin-Bonding Agents[MeSH Terms] OR dentin bonding agent* OR total-etch adhesive* OR total-etch adhesive* OR total-etch OR total-etching OR conventional adhesive OR etch-and-rinse adhesive* OR self-etch adhesive* OR self-etch adhesive* OR self-etch* OR self-etching primer* OR all-in-one adhesive* OR one-bottle adhesive* OR universal adhesive* OR glass-ionomer cements [MeSH Terms] OR glass-ionomer cements OR glass-ionomer cement OR glass polyalkenoate cement* OR resin-modified glass-ionomer cement* OR highly viscous glass-ionomer cement* OR high viscosity glass-ionomer cement AND (((((silver fluoride) OR silver diamine fluoride) OR SDF) OR diamine fluoride). For ISI Web of Science, LILACS and Scopus the following search terms were used: (Silver Diamine Fluoride) OR (Silver Fluoride) AND (Bond Strength).

### Study Selection

Screening of titles and abstracts of all studies were performed to select studies according to the inclusion criteria: in vitro studies that evaluated the bond strength of direct restorative materials (glass-ionomer cement and adhesives/resin composite) to previously SDF-treated dentin. The full-text of potentially eligible studies was assessed. Those which had no control group (dentin without prior application of silver diamine fluoride), assessed root dentin, or used different application protocols of restorative material between the experimental and control groups, were excluded. The reference lists of all included studies were manually screened to retrieve all relevant papers. The studies were selected by two independent reviewers (kappa =0.90), and any disagreement regarding eligibility was solved through discussion and consensus with a third reviewer.

### Data Extraction

The data were extracted according to a predefined protocol using a form in Microsoft Office Excel 2013 (Microsoft; Redmond, WA, USA). For each paper, the following data were systematically extracted: publication year, country, number of teeth per group, type of teeth, silver diamine fluoride and restorative material used, application protocol, bond strength test, dentin condition (sound or caries-affected), type of carious lesion (natural or artificial), bond strength mean values (in MPa) and standard deviations (SD). The authors were contacted via e-mail at least twice to retrieve the bond strengths that were not presented as means and standard deviation. If the authors did not provide this information, the study was not included in the systematic review.

### Risk of Bias Assessment

The risk of bias was based on and adapted from a previous study.^44^ The domains considered were: random sequence generation of the teeth for experimental groups, sample size calculation, the same number of teeth per group, failure mode evaluation, silver diamine fluoride and restorative materials applied following manufacturers’ instructions, materials and testing procedures performed by a single operator, and specimens tested by a blinded operator. If the parameter was described in the text, the study received a “yes,” otherwise, it received a “no” or “unclear” (no information or uncertainty about the potential for bias). The risk of bias was classified according to the sum of “yes” answers received, as follows: 1–3 = high; 4–5 = intermediate; 6–8 = low risk of bias. If needed, authors were contacted via e-mail (at least two attempts were made) for missing or unclear information.

### Data Analysis

Through a random-effects meta-analysis, the pooled-effect estimates were obtained by comparing the standardized mean difference between the bond strengths of SDF treated dentin and control groups separately for each restorative material considered. Subgroup analyses were carried out according to the SDF application protocol (including or excluding the rinsing step after the SDF application time) and dentin condition (sound or caries-affected). p ≤ 0.05 was considered statistically significant (Z-test). For studies that evaluated more than one adhesive, it was necessary to combine the obtained bond strengths (regardless of the etching strategy) into one mean and one standard deviation using a formula suggested by the Cochrane Statistical Guidelines.^17^ Only the immediate bond strengths were considered for analysis. Forest charts were created to illustrate the meta-analysis. Statistical heterogeneity of the treatment effect among studies was assessed using the Cochran Q test and inconsistency (I^2^), with a p-value of 0.1.^17^ All analyses were performed using Review Manager Software 5.3 (The Cochrane Collaboration; Copenhagen, Denmark).

## Results

### Search and Selection

**Fig 1:**
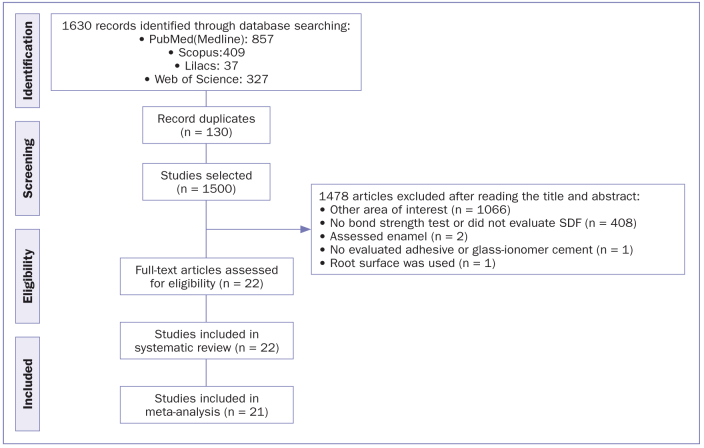
Flowchart diagram of study selection according to the PRISMA statement.

The search strategy identified 1630 potentially eligible studies in all databases. Duplicates were removed, and 1500 studies remained for further examination regarding the inclusion criteria. After screening titles and abstracts, 1478 studies were excluded. With this, 22 studies remained after the full-text assessment. For the meta-analysis, 21 studies were included because one of the studies^54^ did not present means and standard deviation, and the missing data were not provided by the authors through e-mail. A flowchart of the study selection process according to the PRISMA statement^38^ and the reasons for exclusion are shown in Fig 1.

### Descriptive Analysis

Tables 1 and 2 show the descriptive data of the included studies separately by restorative material, glass-ionomer cement, and adhesives. Studies were published between 1993 and 2020, all in English. Almost all of the included studies are from the last eight years, except two^23,54^ evaluating glass-ionomer cement bonding.

**Table 1:** Descriptive data of the included studies – glass-ionomer cement

Study	Country	Number of teeth per group	Silver diamine fluoride (SDF)	SDF protocol*	Restorative material	Type of teeth	Dentin condition	Caries-affected lesion	Bond strength test**
Braz et al, 2020 [3]	Brazil	7	Advantage Arrest < (38%SDF)	Rinsed Not rinsed	Fuji II LC@ Riva Self-Cure&	Permanent teeth	Sound and caries-affected	Artificial (pH-cycling model)	µSBS
François et al, 2020 [10]	France	20	Riva Star& (38% SDF and KI)	Not rinsed	Equia Forte Fil@	Permanent molar	Sound	–	SBS
Gupta et al, 2019 [15]	India	8	Riva Star& (38% SDF and KI)	Rinsed	Gold Label 2 LC@	Permanent molar	Sound	–	SBS
Jiang et al, 2020 [19]	Hong Kong	15	Saforide$$$ (38% SDF)	Not rinse	Ketac-Molar Aplicap #	Permanent molar	Sound and caries-affected	Artificial (microbiological model)	µTBS
Knight et al, 2006 [23]	Australia	10	*** (1.8M SDF and KI)	Not rinsed Rinsed	Fuji VII@	Permanent molar	Sound	–	SBS
Koizumi et al, 2016 [25]	Japan	10	Riva Star& (38% SDF and KI)	Not rinsed	Riva Bond LC&	Permanent molar	Sound	–	µTBS
Ng et al, 2020 [34]	USA	10-12	Advantage Arrest< (38% SDF)	Not Rinsed	Fuiji IX GP Extra Capsule@	Permanent molar	Caries-affected	Artificial (demineralizing solution)	SBS
Puwanawiroj et al, 2018 [42]	Thailand	40	Saforide$$$ (38% SDF)	Rinsed	Fuiji IX GP Extra Capsule@	Primary molar	Caries-affected	Natural	µTBS
Uchil et al, 2020 [49]	India	9	Fagamin$$$$ (38% SDF) Lugol’s solution 10 wt% (KI)%	Rinsed	Gold Label Ligth-Cure Universal@	Primary molar	Caries-affected	Artificial (microbiological model)	µTBS
Wang et al, 2016 [52]	Hong Kong	4	Saforide$$ (38% SDF)	Rinsed	Fuji IX@	Permanent molar	Sound and caries-affected	Artificial (demineralising solution)	µTBS
Yamaga et al, 1993 [54]	Japan	***	Saforide$ (38% SDF)	Not rinsed	Hy-Bond#	Bovine incisor	Sound	–	SBS
Zhao et al, 2019 [55]	Hong Kong	20	Riva Star& (38% SDF and KI) Saforide$$$ (38% SDF)	Rinsed Not rinsed	Ketac-Molar#	Permanent	Caries-affected	Artificial (demineralising solution)	SBS

* SDF protocol: not rinsed or rinsed after the waiting time;^a^ elapsed time between the placement of SDF and restoration; **SBS: shear bond strength; µTBS: microtensile bond strength; µSBS: microshear bond strength; *** not given; $ Toyo Pharmaceutical, Tokyo, Japan; # Shofu, Tokyo, Japan; @ GC, Tokyo, Japan; # 3M Oral Care, St Paul, MN, USA; & SDI, Bayswater, Victoria, Australia; $$ Morita, Osaka, Japan; $$$ Bee Brand Medico Dental, Osaka, Japan; $$$$ Tedequim Company, Córdoba, Argentina; < Elevate Oral Care, West Palm Beach, FL, USA; % Nice Chemicals, Kochi, India.

**Table 2:** Descriptive data of the included studies – adhesives

Study	Country	Number of teeth per group	Silver diamine fluoride (SDF)	SDF protocol*	Restorative material	Type of teeth	Dentin condition	Type of caries-affected lesion	Bond strength test**
Firouzmandi et al, 2020 [9]	Iran	12	Ancarie Cariostaticº (30% SDF)	Rinsed	Adper Single Bond 2%	Permanent molar	Sound and caries-affected	Natural	µSBS
Ko et al, 2020 [24]	Hong Kong	16	Saforide$$ (38%SDF) Saforide RC$$ (3.8% SDF)	Rinsed	Clearfil SE Bond#	Permanent molar	Sound	–	µTBS
Koizumi et al, 20016 [25]	Japan	10	Riva Star& (38% SDF and KI)	Not rinsed	Optibond FL Optibond Versa Clearfil Liner Bond#	Permanent molar	Sound	–	µTBS
Kucukylmaz et al, 2016 [26]	Turkey	8	Saforide$ (38% SDF)	Not rinsed	Clearfil SE Bond#	Permanent molar	Sound and caries-affected	Artificial (pH-cycling model)	µTBS
Lutgen et al, 2018 [28]	USA	10	Advantage Arrest< (38% SDF)	Not rinsed Rinsed	Clearfil SE Bond 2# Scotchbond Universal%	Permanent molar	Sound	–	µSBS
Markham et al, 2020 [29]	USA	15	Advantage Arrest < (38% SDF)	Rinsed	Scotchbond Universal% Prime & Bond NT > G-Premio Bond £	Permanent molar	Sound	–	SBS
Quock et al, 2012 [43]	USA	7	Saforide$ (38% SDF) Ancarie Cariostaticº (12% SDF)	Rinsed	Peak SE@ Peak LC@	Permanent molar	Sound	–	µTBS
Selvaraj et al, 2016 [45]	India	18	Riva Star& (38% SDF and KI)	Rinsed	Adper Single Bond 2% Adper Easy One%	Permanent molar	Sound	–	µSBS
Siqueira et al, 2020 [46]	Brazil	5	Riva Star& (38% SDF and KI) Cariestop? (12% SDF)	Rinsed	Clearfil Universal Bond Quick# Scotchbond Universal%	Permanent molar	Caries-affected	Artificial (microbiological model)	µTBS
Van Duker et al, 2019 [50]	USA	10	Advantage Arrest< (38% SDF) Saturated solution of KI	Rinsed	Scotchbond Universal%	Permanent molar	Caries-affected	Artificial (demineralising solution)	µTBS
Wu et al, 2016 [53]	USA	12	Saforide$ (38% SDF)	Rinsed	Prime & Bond NT >	Primary molar	Sound	–	µTBS

* SDF protocol: not rinsed or rinsed after the waiting time; ª elapsed time between the placement of SDF and restoration; **µTBS: microtensile bond strength; µSBS: microshear bond strength; º Maquira Dental Products, Maringa, PR, Brazil; $ Toyo Seiyaku Kansei, Osaka, Japan; $$Bee Brand Medico Dental, Osaka, Japan; @ Ultradent, South Jordan, UT, USA; & SDI, Bayswater, Victoria, Australia; ^ Kerr, Orange, CA, USA; # Kuraray Noritake Dental, Tokyo, Japan; % 3M Oral Care, St Paul, MN, USA; ? Biodinâmica, RJ, Brazil; < Elevate Oral Care, West Palm Beach, FL, USA; > Dentsply Caulk, Milford, DE, USA; £ GC, Tokyo, Japan.

For glass-ionomer cement, 12 studies were included.^3,10,15,19,23,25,34,42,49,52,55^ The studies come mainly from 3 countries/regions – Japan, India, and Hong Kong. SDF concentrations of 30% or 38% associated with potassium iodide solution were evaluated. The majority of the studies evaluated glass-ionomer cement modified by resin or high viscosity; only three used conventional glass-ionomer cement.^3,23,52^ Five studies evaluated the bonding of glass-ionomer cement to sound dentin,^10,15,23,34^ four considered caries-affected dentin,^34,42,49,55^ and three evaluated the two substrate conditions.^3,19,52^ Of the studies that assessed caries-affect dentin, most used artificial lesions; only one study^42^ used natural lesions. The majority assessed permanent teeth, and only two studies used primary molars.^42,49^ The shear bond strength test was the most frequently employed mechanical method, followed by the microtensile bond strength test.

For adhesives, eleven studies were included.^9,24-26,28,29,43,45,46,50,53^ The studies come mainly from the USA. The majority of studies used SDF concentrations of 30% or 38%, associated with potassium iodide solution. Only two studies^43,46^ evaluated both SDF 38% and SDF 12%, and one study^24^ assessed SDF 38% and 3.8%. Therefore, data based on concentrations of 12% and 3.8% were not considered. Sound dentin was the bonding substrate considered in most studies; only four studies^9,26,46,50^ evaluated the bond strength to caries-affected dentin. Of the studies that assessed caries-affect dentin, most used artificial lesions and only one study^9^ used natural lesions. Only one study considered bonding to primary dentin.^53^ The microtensile bond strength test was the most commonly used mechanical test.

### Meta-Analysis

#### Glass-ionomer cement

**Fig 2:**
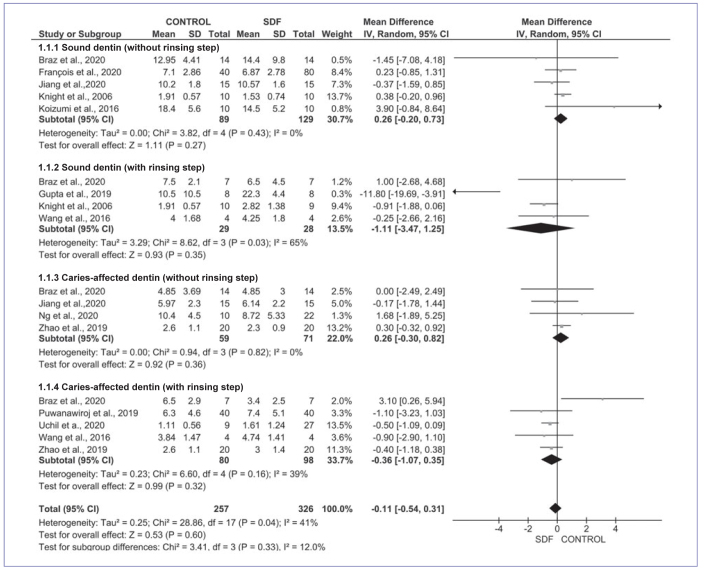
Meta-analysis findings comparing the bond strength of glass-ionomer cement to SDF treated (SDF) and untreated (control) dentin according to SDF protocol (with or without rinsing step) and dentin condition (sound or caries-affected).

Figure 2 shows the results for the meta-analysis considering glass-ionomer cement. No significant difference was found between control and SDF groups (Z = 0.53; p =0.60) in the overall meta-analysis, with moderate heterogeneity (chi-squared test; p =0.04; I^2^ =41 %). SDF pretreatment does not affect the bond strength to sound dentin, with (Z = 0.93; p = 0.35) or without the rinsing step (Z = 1.11; p = 0.27). The data showed heterogeneity for rinsing subgroups (chi-squared test; p = 0.03; I^2^ =65 %) and no heterogeneity for subgroups without a rinsing step (chi-squared test; p = 0.43; I^2^=0%). Similarly, pretreatment with SDF did not jeopardize the bond strength to caries-affected dentin, with (Z = 0.99, p = 0.32) or without a rinsing step (Z = 0.92, p = 0.36). The data show moderate heterogeneity for rinsing subgroups (chi-squared test; p = 0.16; I^2^ = 39%) and no heterogeneity for subgroups without rinsing (chi-squared test; p = 0.82; I^2^ = 0%).

#### Adhesives

**Fig 3:**
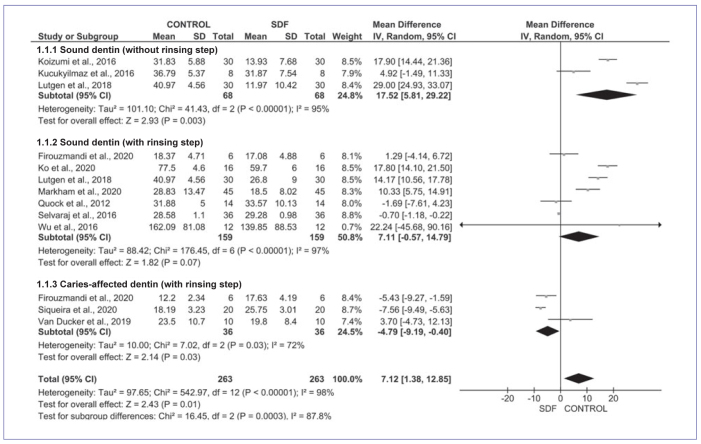
Meta-analysis findings comparing the bond strength of adhesives to SDF treated (SDF) and untreated (control) dentin according to SDF protocol (with or without rinsing step) and dentin condition (sound or caries-affected).

Figure 3 shows the results of meta-analysis for adhesives. SDF applied prior to the adhesive significantly impaired the bond strength to dentin in overall meta-analysis (Z = 2.43; p = 0.01) and in the sound dentin subgroup without rinsing (Z = 2.93; p<0.01). The data were heterogeneous (chi-squared test; p < 0.01; I^2^ =98% and p < 0.01; I^2^ =95%). The rinsing step eliminated the negative effect of SDF application in sound dentin (Z = 1.82; p = 0.07) and increased the bonding of adhesives to caries-affected dentin (Z = 2.14, p = 0.03). Data were heterogeneous in both sound and caries-affected dentin, with rinsing step subgroup analysis (chi-squared test; p < 0.01; I^2^=97%; chi-squared test; p = 0.1; I^2^=72%).

#### Risk of bias

The results of the risk-of-bias assessment are described in Table 3. One study could not have its risk of bias assessed,^50^ as it was not obtained in the full version, and some domains could not be evaluated. The majority of the included studies presented intermediate (10 studies) or high (8 studies) risk of bias. The parameters that most often received “no” were: the description of sample size calculation, a single operator during the specimen preparation, and operator blinded during the tests.

**Table 3:** Risk of bias assessment for each included study

Study	Random sequence	Sample size calculation	Same number of teeth per group	Failure mode evaluation	SDF application protocol according manufactures	Restorative material application protocol according manufactures	Single operator	Blinded operator	Risk of bias
Glass-ionomer cement									
Braz et al, 2020 [3]	Yes	Yes	Yes	Yes	Yes	Yes	Yes	No	Low
François et al, 2020 [10]	Yes	No	Yes	Yes	Yes	Yes	No	No	Medium
Gupta et al, 2019 [15]	No	No	Yes	No	Yes	Yes	No	No	High
Jiang et al, 2020 [19]	Yes	No	Yes	Yes	Yes	Yes	No	No	Medium
Knight et al, 2006 [23]	No	No	No	No	Unclear	Unclear	No	No	High
Koizumi et al, 2016 [25]	Yes	No	Yes	Yes	Yes	Yes	No	No	Medium
Ng et al, 2020 [34]	Yes	No	No	No	Yes	Yes	No	No	High
Puwanawiroj et al, 2018 [42]	Yes	No	Yes	Yes	Unclear	Unclear	No	No	High
Uchil et al, 2020 [49]	Yes	Yes	Yes	Yes	Unclear	Yes	No	No	Medium
Wang et al, 2016 [52]	Yes	No	Yes	Yes	Yes	Unclear	No	No	Medium
Yamaga et al, 1993 [54]	No	No	Yes	Yes	No	No	No	No	High
Zhao et al, 2019 [55]	Yes	No	Yes	Yes	Unclear	Unclear	No	No	High
Adhesives									
Firouzmandi et al, 2020 [9]	Yes	No	Yes	Yes	Unclear	Yes	No	No	Medium
Ko et al, 2020 [24]	Yes	No	Yes	Yes	Unclear	Yes	No	No	Medium
Koizumi et al, 20016 [25]	Yes	No	Yes	Yes	Yes	Yes	No	No	Medium
Kucukylmaz et al, 2016 [26]	Yes	No	Yes	Yes	Unclear	Unclear	No	No	High
Lutgen et al, 2018 [28]	No	No	Yes	Yes	Yes	Yes	No	No	Medium
Markham et al, 2020 [29]	No	No	Yes	Yes	Unclear	Yes	No	No	High
Quock et al, 2012 [43]	Yes	No	Yes	Yes	No	Yes	No	No	Medium
Selvaraj et al, 2016 [45]	Yes	No	Yes	Yes	No	Yes	No	No	Medium
Siqueira et al, 2020 [46]	Yes	Yes	Yes	Yes	Yes	Yes	Yes	No	Low
Wu et al, 2016 [53]	No	No	Yes	Yes	Unclear	Yes	No	No	High

## Discussion

The present study updates a previous systematic review and meta-analysis on the influence of silver diamine fluoride application on dentin bond strength of glass-ionomer cement and adhesives.^11^ The first review^11^ included 11 studies, the majority of them evaluating SDF pretreatment on sound dentin, so that an independent analysis with data of caries-affected dentin could not be investigated. This updated systematic review, conducted by the same research group, included 22 studies; 10 studies that evaluated caries-affected dentin substrate were eligible for this review (6 new studies); this is a more relevant substrate in daily clinical practice. Another systematic review was recently published, but without including the total of studies considered in this review.^20^

According to our previous systematic review,^11^ the effect of SDF application on dentin bonding was material-dependent, as it does not influence the bonding of glass-ionomer cement but can impair the bond strength of adhesives if SDF is not rinsed after application. Therefore, new subgroup meta-analyses were performed considering studies that assessed the SDF effect in caries-affected dentin. As in the previous review, SDF pretreatment in caries-affected dentin does not impair the bond strength of glass-ionomer cement. Likewise, SDF application followed by rinsing, does not jeopardize adhesives’ bond strength. Therefore, considering the obtained results, the hypothesis of this updated review was partially accepted.

The present findings show that regardless of dentin condition (sound or caries-affected), the bonding of glass-ionomer cement was not affected by SDF pretreatment. This may be explained by the bonding mechanism of glass-ionomer cement, which is based on a chemical reaction between polyacrylic acid from glass ionomer and calcium ions mainly from hydroxyapatite.^14^ The SDF protocol (with or without the rinsing step) did not influence dentin bond strength to either substrate. However, even in studies that did not carry out the rinsing step immediately after SDF application, a conditioner, such as polyacrylic acid,^3,10,19,25^ was applied before restoration. Thus, the rinsing step or the application of polyacrylic acid can be necessary for proper adhesion of glass-ionomer cement to SDF-treated dentin to eliminate the silver precipitate excess and increase the ion exchange for an acid-base reaction.

In contrast to the glass-ionomer cement results, the bond strength of adhesives to dentin could be impaired by SDF pretreatment. The silver precipitate formed on the dentin surface and in dentin tubules could adversely affect the bonding of the adhesive, as the bonding mechanism of adhesives is based on micromechanical retention and hybrid layer formation in dentin.^4,16^ As in the first review, this update considered the adhesives in the same group, regardless of the etching strategy (etch-and-rinse or self-etch), as in other reviews,^2,7,41^ considering that the main goal was to evaluate the influence of SDF on dentin bonding.

The negative effect of SDF on the bonding of adhesives was eliminated when rinsing was performed, even in caries-affected dentin. The worst result was expected in caries-affected dentin due to the chemical and morphological differences^1,33^ as well as more silver precipitating onto demineralized dentin,^22^ but this was not confirmed in this systematic review. On the contrary, pretreatment with SDF followed by rinsing could increase adhesives’ bonding to caries-affected dentin. Only one study^26^ assessed prior SDF application on caries-affected dentin without the rinsing step, so this subgroup analysis could not be performed. Even so, according to this earlier study,^26^ SDF application jeopardized dentin bond strength. Immediately after SDF application, rinsing can eliminate the excess of silver precipitate from peritubular and intertubular dentin,^28^ favoring adhesion. Besides, SDF can remineralize the caries-affected dentin,^56^ improving the mechanical properties of this altered substrate. However, this finding is based on only three studies;^9,46,50^ thus, more investigations evaluating the bonding mechanism of adhesives to SDF-treated caries-affected dentin are necessary to confirm this result.

The application of SDF to prevent recurrent caries is a new and off-label approach; therefore, few studies evaluating its effect on bond strength of restorative materials are available, which explains the relatively low number of eligible studies. However, it is essential to note that shortly after the publication of the first systematic review,^11^ twice as many articles could now be included in this update, demonstrating the growing interest in the use of SDF. Despite the considerable increase in the number of studies, only one new study evaluating primary teeth was included.^49^ Thus, although SDF is most commonly used for arresting caries in primary teeth,^5^ few studies assessed the influence of prior SDF application on the bond strength of restorative materials in these teeth (two assessed glass-ionomer cement^42,49^ and only one considered an adhesive).^53^ Hence, a separate meta-analysis cannot be performed yet. Considering that bond strength evaluations can measure one specific parameter, controlling the other variables, more laboratory investigations should be conducted to evaluate the effect of SDF on bonding to primary dentin.

SDF can inhibit matrix metalloproteinases (MMPs), thus avoiding dentin collagen degradation.^30,31^ It is known that the intrinsic degradation (proteolysis) of collagen fibers in dentin by enzymes such as MMPs can compromise the bonding interface, decreasing the bond strength of restorative materials in the long term.^37,39^ The use of MMP inhibitors has been considered an effective strategy to improve the longevity of adhesive restorations.^37,48^ However, only one included study evaluated the bond strength of an etch-and-rinse adhesive to SDF-treated dentin after 6 months of water storage;^9^ therefore, the influence of aging could not be evaluated through a meta-analysis. That study^9^ found that prior SDF application on sound dentin limited the effect of water storage on the bond strength; however, on caries-affected dentin, a significant reduction of bond strength after water storage was shown in the SDF-treated group. Therefore, long-term studies are needed to determine the effect of silver diamine fluoride on bond strength after aging.

The present systematic review assessed the influence of SDF on bonding, mainly in concentrations of 30% and 38%, as previous studies demonstrated that products with the highest concentrations are more effective in arresting caries lesions.^12,13^ Only two studies assessed 12% SDF.^43,46^ Moreover, 9 studies evaluated dentin pretreatment with 38% SDF associated with potassium iodide (KI) before restoration placement.^10,15,23,25,45,46,49,50,55^ The subsequent application of KI is suggested to minimize the inherent disadvantage of SDF turning treated areas dark.^40^ A previous study^57^ suggests that the association SDF+KI is not as effective as SDF alone in preventing secondary caries, while another^23^ reported that this association was more effective in inhibiting the migration of *Streptococcus mutans* through dentin than SDF alone. Thus, there are doubts regarding the effectiveness of SDF with KI for caries prevention.

As found in this systematic review, high heterogeneity is a common finding in meta-analyses of laboratory studies,^6,27,44,47^ and in this case, may have been influenced by the SDF application protocol, restorative material, different methods of producing caries-affected dentin and bond strength testing. Besides, most studies included presented a high or intermediate risk of bias. Although there is a guideline for conducting and reporting in vitro studies on dental materials,^8^ it seems that it has not been commonly used, so this finding in systematic reviews of laboratory studies is common.^6,27,47^

This systematic review evaluated the bond strength of glass-ionomer cement and adhesives to silver diamine fluoride-treated dentin. Bond strength tests are commonly used to evaluate restorative materials to predict their performance and the influencing variables – such as prior application of SDF. Although the relationship between in vitro studies and clinical performance is difficult to establish,^51^ a material’s adhesive ability is an indicator of restoration longevity; superior laboratory performance is probably indicative of better clinical performance.^35^ Nevertheless, the results of in vitro studies should ideally be confirmed by long-term laboratory studies and randomized clinical trials, evaluating not only the effect of SDF pretreatment on bonding but also considering interface integrity, secondary caries, and staining. At the moment of this review, there was only one randomized clinical trial with 24 months of follow-up which evaluated the prior application of SDF on cavitated dentin caries lesions in primary teeth before atraumatic restorative treatment (ART).^21^ That clinical trial found that SDF did not jeopardize the success rate of the restorations.

The present systematic review pointed out that pretreatment with silver diamine fluoride does not influence the bond strength of glass-ionomer cement to dentin, regardless of whether it is sound or caries-affected. In contrast, SDF application can impair the bonding of adhesives. However, rinsing after SDF application seems to eliminate this adverse effect in sound dentin and improved the bond strength to caries-affected dentin.

## Conclusion

Based on the results of this systematic review and meta-analysis of in vitro studies, it can be concluded that the SDF pretreatment does not jeopardize the bonding of glass-ionomer cement to dentin. The same is valid for adhesives only if a rinsing step after SDF application is performed, which can even improve the adhesion of this restorative material to caries-affected dentin.

## Clinical relevance:

Dentin pretreatment with silver diamine fluoride does not affect the adhesion of glass-ionomer cements. If a rinsing step after SDF application is not carried out, the bonding of the adhesive to dentin may be compromised.

## References

[ref1] Angker L, Nockolds C, Swain MV, Kilpatrick N (2004). Quantitative analysis of the mineral content of sound and carious primary dentin using BSE imaging. Arch Oral Biol.

[ref2] Bohrer TC, Fontana PE, Lenzi TL, Soares F, Rocha RO (2018). Can endodontic irrigating solutions influence the bond strength of adhesives to coronal dental substrates? A systematic review and meta-analysis of in vitro studies. J Adhes Dent.

[ref3] Braz PVF, Dos Santos AF, Leal SC, Pereira PN, Ribeiro APD (2020). The effect of silver diamine fluoride and cleaning methods on bond strength of glass-ionomer cements to caries-affected dentin. Am J Dent.

[ref4] Cardoso MV, de Almeida Neves A, Mine A, Coutinho E, Van Landuyt K, De Munck J, Van Meerbeek B (2011). Current aspects on bonding effectiveness and stability in adhesive dentistry. Aust Dent J.

[ref5] Chibinski AC, Wambier LM, Feltrin J, Loguercio AD, Wambier DS, Reis A (2017). Silver diamine fluoride has efficacy in controlling caries progression in primary teeth: A systematic review and meta-analysis. Caries Res.

[ref6] Cuevas-Suárez CE, da Rosa WLO, Lund RG, da Silva AF, Piva E (2019). Bonding performance of universal adhesives: An updated systematic review and meta-analysis. J Adhes Dent.

[ref7] De Carvalho MFF, Leijôto-Lannes ACN, Rodrigues MCN, Nogueira LC, Ferraz NKL, Moreira AN, Yamauti M, Zina LG, Magalhães CS (2018). Viability of bovine teeth as a substrate in bond strength tests: A systematic review and meta-analysis. J Adhes Dent.

[ref8] Faggion CM Jr (2012). Guidelines for reporting pre-clinical in vitro studies on dental materials. J Evid Based Dent Pract.

[ref9] Firouzmandi M, Mohaghegh M, Jafarpisheh M (2020). Effect of silver diamine fluoride on the bond durability of normal and carious dentin. J Clin Exp Dent.

[ref10] François P, Greenwall-Cohen J, Le Goff S, Ruscassier N, Attal JP, Dursun E (2020). Shear bond strength and interfacial analysis of high-viscosity glass-ionomer cement bonded to dentin with protocols including silver diammine fluoride. J Oral Sci.

[ref11] Fröhlich TT, Rocha RO, Botton G (2020). Does previous application of silver diammine fluoride influence the bond strength of glass-ionomer cement and adhesive systems to dentin? Systematic review and meta-analysis. Int J Paediatr Dent.

[ref12] Fung MHT, Duangthip D, Wong MCM, Lo ECM, Chu CH (2016). Arresting dentin caries with different concentration and periodicity of silver diamine fluoride. JDR Clin Trans Res.

[ref13] Fung MHT, Duangthip D, Wong MCM, Lo ECM, Chu CH (2018). Randomized clinical trial of 12% and 38% silver diamine fluoride treatment. J Dent Res.

[ref14] Gjorgievska ES, Nicholson JW, Apostolska SM, Coleman NJ, Booth SE, Slipper IJ, Mladenov MI (2013). Interfacial properties of three different bioactive dentin substitutes. Microsc Microanalysis.

[ref15] Gupta J, Thomas MS, Radhakrishna M, Srikant N, Ginjupalli K (2019). Effect of silver diamine fluoride-potassium iodide and 2% chlorhexidine gluconate cavity cleansers on the bond strength and microleakage of resin-modified glass-ionomer cement. J Conserv Dent.

[ref16] Hashimoto M, Ohno H, Endo K, Kaga M, Sano H, Oguchi H (2000). The effect of hybrid layer thickness on bond strength: demineralized dentin zone of the hybrid layer. Dent Mater.

[ref17] Higgins JPT, Green S editors (2011). Cochrane handbook for Systematic Reviews of Interventions. Version 5.1.0 [updated March 2011]. The Cochrane Collaboration.

[ref18] Isolan CP, Sarkis-Onofre R, Lima GS, Moraes RR (2018). Bonding to sound and caries-affected dentin: A systematic review and meta-analysis. J Adhes Dent.

[ref19] Jiang M, Mei ML, Wong M, Chu CH, Lo E (2020). Influence of silver diamine fluoride treatment on the microtensile bond strength of glass-ionomer cement to sound and carious dentin. Oper Dent.

[ref20] Jiang M, Mei ML, Wong MCM, Chu CH, Lo ECM (2020). Effect of silver diamine fluoride solution application on the bond strength of dentin to adhesives and to glass-ionomer cements: a systematic review. BMC Oral Health.

[ref21] Jiang M, Wong MCM, Chu CH, Dai L, Lo ECM (2020). A 24-month randomized controlled trial on the success rates of restoring untreated and SDF-treated dentin caries lesions in primary teeth with the ART approach. J Dent.

[ref22] Knight GM, McIntyre JM, Craig GG, Mulyani, Zilm PS, Gully NJ (2007). Differences between normal and demineralized dentin pretreated with silver fluoride and potassium iodide after an in vitro challenge by Streptococcus mutans. Aust Dent J.

[ref23] Knight GM, McIntyre JM, Mulyani (2006). The effect of silver fluoride and potassium iodide on the bond strength of auto cure glass-ionomer cement to dentin. Aust Dent J.

[ref24] Ko AK, Matsui N, Nakamoto A, Ikeda M, Nikaido T, Burrow MF, Tagami J (2020). Effect of silver diammine fluoride application on dentin bonding performance. Dent Mater J.

[ref25] Koizumi H, Hamana HH, Burrow MF (2016). Effect of a silver diamine fluoride and potassium iodide-based desensitizing and cavity cleaning agent on bond strength to dentin. Int J Adhes Adhes.

[ref26] Kucukyilmaz E, Savas S, Akcay M, Bolukbasi B (2016). Effect of silver diamine fluoride and ammonium hexafluorosilicate applications with and without Er:YAG laser irradiation on the microtensile bond strength in sound and caries-affected dentin. Lasers Surg Med.

[ref27] Lenzi TL, Gimenez T, Tedesco TK, Mendes FM, Rocha Rde O, Raggio DP (2016). Adhesive systems for restoring primary teeth: a systematic review and meta-analysis of in vitro studies. Int J Paediatr Dent.

[ref28] Lutgen P, Chan D, Sadr A (2018). Effects of silver diammine fluoride on bond strength of adhesives to sound dentin. Dent Mater J.

[ref29] Markham MD, Tsujimoto A, Barkmeier WW, Jurado CA, Fischer NG, Watanabe H, Baruth AG, Latta MA, Garcia-Godoy F (2020). Influence of 38% silver diamine fluoride application on bond stability to enamel and dentin using universal adhesives in self-etch mode. Eur J Oral Sci.

[ref30] Mei ML, Ito L, Cao Y, Li QL, Lo EC, Chu CH (2013). Inhibitory effect of silver diamine fluoride on dentin demineralisation and collagen degradation. J Dent.

[ref31] Mei ML, Li QL, Chu CH, Yiu CK, Lo EC (2012). The inhibitory effects of silver diamine fluoride at different concentrations on matrix metalloproteinases. Dent Mater.

[ref32] Mei ML, Zhao IS, Ito L, Lo EC, Chu CH (2016). Prevention of secondary caries by silver diamine fluoride. Int Dent J.

[ref33] Nakajima M, Kitasako Y, Okuda M, Foxton RM, Tagami J (2005). Elemental distributions and microtensile bond strength of the adhesive interface to normal and caries-affected dentin. J Biomed Mater Res B Appl Biomater.

[ref34] Ng E, Saini S, Schulze KA, Horst J, Le T, Habelitz S (2020). Shear bond strength of glass-ionomer cement to silver diamine fluoride-treated artificial dentinal caries. Pediatr Dent.

[ref35] Opdam NJ, van De Sande FH, Bronkhorst E, Cenci MS, Bottenberg P, Pallesen U, Gaengler P, Lindberg A, Huysmans MC, Van Dijken JW (2014). Longevity of posterior composite restorations: a systematic review and meta-analysis. J Dent Res.

[ref36] Osama MF, Weaam AD, Mona TA, Bashaer A, Faris SB, Ghada F, Luluah KA, Sumaya MN (2018). The effect of silver diamine fluoride in preventing secondary caries in primary teeth: In-vitro study. EC Dent Sci.

[ref37] Osorio R, Yamauti M, Osorio E, Ruiz-Requena ME, Pashley D, Tay F, Toledano M (2011). Effect of dentin etching and chlorhexidine application on metalloproteinase-mediated collagen degradation. Eur J Oral Sci.

[ref38] Page MJ, McKenzie JE, Bossuyt PM, Boutron I, Hoffmann TC, Mulrow CD et al (2021). The PRISMA 2020 statement: an updated guideline for reporting systematic reviews. BMJ.

[ref39] Pashley DH, Tay FR, Breschi L, Tjäderhane L, Carvalho RM, Carrilho M, Tezvergil-Mutluay A (2011). State of the art etch-and-rinse adhesives. Dent Mater.

[ref40] Patel J, Anthonappa RP, King NM (2018). Evaluation of the staining potential of silver diamine fluoride: in vitro. Int J Paediatr.

[ref41] Pires CW, Soldera EB, Bonzanini LIL, Lenzi TL, Soares FZM, Montagner AF, Rocha RO (2018). Is adhesive bond strength similar in primary and permanent teeth? A systematic review and meta-analysis. J Adhes Dent.

[ref42] Puwanawiroj A, Trairatvorakul C, Dasanayake AP, Auychai P (2018). Microtensile bond strength between glass-ionomer cement and silver diamine fluoride-treated carious primary dentin. Pediatr Dent.

[ref43] Quock RL, Barros JA, Yang SW, Patel SA (2012). Effect of silver diamine fluoride on microtensile bond strength to dentin. Oper Dent.

[ref44] Sarkis-Onofre R, Skupien JA, Cenci MS, Moraes RR, Pereira-Cenci T (2014). The role of resin cement on bond strengthof glass-fiber posts luted into root canals: a systematicreview and meta-analysis of in vitro studies. Oper Dent.

[ref45] Selvaraj K, Sampath V, Sujatha V, Mahalaxmi S (2016). Evaluation of microshear bond strength and nanoleakage of etch-and-rinse and self-etch adhesives to dentin pretreated with silver diamine fluoride/potassium iodide: An in vitro study. Indian J Dent Res.

[ref46] Siqueira FSF, Morales LAR, Granja MCP, De Melo BO, Monteiro-Neto V, Reis A, Cardenas AFM, Loguercio AD (2020). Effect of silver diamine fluoride on the bonding properties to caries-affected dentin. J Adhes Dent.

[ref47] Soares FZM, Follak A, da Rosa LS, Montagner AF, Lenzi TL, Rocha RO (2016). Bovine tooth is a substitute for human tooth in bond strength studies: A systematic review and meta-analysis of in vitro studies. Dent Mater.

[ref48] Stanislawczuk R, Reis A, Loguercio AD (2011). A 2-year in vitro evaluation of a chlorhexidine-containing acid on the durability of resin-dentin interfaces. J Dent.

[ref49] Uchil SR, Suprabha BS, Suman E, Shenoy R, Natarajan S, Rao A (2020). Effect of three silver diamine fluoride application protocols on the microtensile bond strength of resin-modified glass-ionomer cement to carious dentin in primary teeth. J Indian Soc Pedod Prev Dent.

[ref50] Van Duker M, Hayashi J, Chan DC, Tagami J, Sadr A (2019). Effect of silver diamine fluoride and potassium iodide on bonding to demineralized dentin. Am J Dent.

[ref51] Van Meerbeek B, Peumans M, Poitevin A, Mine A, Van Ende A, Neves A, De Munck J (2010). Relationship between bond-strength tests and clinical outcomes. Dent Mater.

[ref52] Wang AS, Botelho MG, Tsoi JKH, Matinlinna JP (2016). Effects of silver diamine fluoride on microtensile bond strength of GIC to dentin. Int J Adhes Adhes.

[ref53] Wu DI, Velamakanni S, Denisson J, Yaman P, Boynton JR, Papagerakis P (2016). Effect of silver diamine fluoride (SDF) application on microtensile bonding strength of dentin in primary teeth. Pediatr Dent.

[ref54] Yamaga M, Koide T, Hieda T (1993). Adhesiveness of glass-ionomer cement containing tannin-fluoride preparation (HY agent) to dentin – an evaluation of adding various ratios of HY agent and combination with application diammine silver fluoride. Dent Mater J.

[ref55] Zhao IS, Chu S, Yu OY, Mei ML, Chu CH, Lo ECM (2019). Effect of silver diamine fluoride and potassium iodide on shear bond strength of glass-ionomer cements to caries-affected dentin. Int Dent J.

[ref56] Zhao IS, Gao SS, Hiraishi N, Burrow MF, Duangthip D, Mei ML, Lo EC, Chu CH (2018). Mechanisms of silver diamine fluoride on arresting caries: a literature review. Int Dent J.

[ref57] Zhao IS, Mei ML, Burrow MF, Lo EC, Chu CH (2017). Effect of silver diamine fluoride and potassium iodide treatment on secondary caries prevention and tooth discolouration in cervical glass-ionomer cement restoration. Int J Mol Sci.

